# The Cosmos and Crystal Connection: What We Can Learn About Data Compression

**DOI:** 10.1063/4.0001071

**Published:** 2025-10-27

**Authors:** Kimberly M. Horvat, Herbert J. Bernstein, Alexei S. Soares, Jean Jakoncic

**Affiliations:** 1 Stony Brook University, Stony Brook, NY, USA; 2 Brookhaven National Laboratory, Upton, NY, USA; 3 Fresh Pond Research Institute, Cambridge, MA, USA

## Abstract

We use both lossy and lossless compression methods to reduce large amounts of digital data, making it easier to store and manage. Lossy and lossless compression is frequently used in astronomy, to condense the large amount of data collected by instruments namely, the James Webb Space Telescope and the Hubble Space Telescope. Similarly to diffraction images, astronomical images have large areas of empty space since stellar objects are very bright, yet show very small in images, which after a while, takes up a needless amount of limited digital storage space. Lossy compression achieves higher compression ratios but sacrifices some data quality in the process, while lossless compression preserves the data’s original form, but results in less storage savings. This work, conducted at Brookhaven National Laboratory’s National Synchrotron Light Source-II at the AMX and FMX macromolecular beam lines, aimed to evaluate the effectiveness of the lossy compression method known as Wavelet Compression.

Wavelet compression is a method of data compression that uses wavelet transforms to reduce the size of data while preserving its essential features. Crystals are repetitive molecular assemblies that create diffraction patterns when exposed to X-rays, while detectors capture the amplitude of diffraction pattern spots (reflections). Similar to astronomical images, diffraction images contain large areas of empty space. This unnecessary data can quickly consume limited digital storage. By studying and optimizing compression methods, we not only improve data management but also advancements in various scientific disciplines.

